# Combined Transplantation of Mesenchymal Stem Cells and Endothelial Colony-Forming Cells Accelerates Refractory Diabetic Foot Ulcer Healing

**DOI:** 10.1155/2020/8863649

**Published:** 2020-09-09

**Authors:** Liling Zhao, Zi Guo, Ke Chen, Wenjun Yang, Xinxing Wan, Pingyu Zeng, Honghui He, Yufang Luo, Qian Xiao, Zhaohui Mo

**Affiliations:** ^1^Department of Endocrinology, Third Xiangya Hospital of Central South University, Changsha, Hunan Province, China 410013; ^2^Center for Experimental Medicine, Third Xiangya Hospital of Central South University, Changsha, Hunan Province, China 410013

## Abstract

**Background:**

This study is aimed at investigating the effect of combined transplantation of umbilical cord mesenchymal stem cells (UCMSCs) and umbilical cord blood-derived endothelial colony-forming cells (ECFCs) on diabetic foot ulcer healing and at providing a novel therapy for chronic diabetic foot ulcer.

**Methods:**

We reported the treatment of refractory diabetic foot ulcers in twelve patients. Among them, five patients had two or more wounds; thus, one wound in the same patient was treated with cell injection, and other wounds were regarded as self-controls. The remaining seven patients had only one wound; therefore, the difference between the area of wound before and after treatment was estimated. The UCMSCs and ECFCs were injected into the wound along with topically applied hyaluronic acid (HA).

**Results:**

In this report, we compared the healing rate of multiple separate wounds in the same foot of the same patient: one treated with cell injection combined with topically applied HA-based hydrogel and was later covered by the hydrocolloid dressings, while the self-control wounds were only treated with conventional therapy and covered by the hydrocolloid dressings. The wound underwent cell injection showed accelerated healing in comparison to control wound within the first week after treatment. In other diabetic patients with only one refractory wound, the healing rate after cell transplantation was significantly faster than that before injection. Two large wounds healed without needing skin grafts after combination therapy of cell injection and HA. After four weeks of combination treatment, wound closure was reached in six patients, and the wounds of the other six patients were significantly reduced in size.

**Conclusions:**

Our study suggests that the combination of UCMSCs, ECFCs, and HA can safely synergize the accelerated healing of refractory diabetic foot ulcers.

## 1. Introduction

Diabetic foot ulcers (DFUs) are common diabetic complications with a global prevalence of 6.3% [1]. DFU healing usually takes weeks or even months, and 14–24% of ulcers will end up in amputations [2]. Thus, measures to accelerate DFU healing are urgent for reducing the high cost and heavy burden of DFU management. Wound closure rate within the first four weeks of treatment is a robust predictor of healing outcome [3, 4]. Therefore, acceleration of epithelialization across the wound surface remains a principal aim in the management of DFUs.

Mesenchymal stem cell (MSC) therapy is a promising approach for DFUs. Previous studies have demonstrated that either injections around the ulcers [5–7] or topical applications of bone marrow-derived MSCs delivered in a fibrin spray [8] could significantly improve wound healing in diabetic patients. Hence, alternative sources of MSC have been exploited, such as the umbilical cord, which is an extraembryonic tissue discarded at birth. Umbilical cord MSCs are a primitive population of stromal cells, and their proliferation rate and *in vitro* expansion capacity are superior to those of adult tissue-derived MSC partially due to the expression of telomerase [9–11]. In addition, a stem cell transplantation does not elicit graft rejection, even in xenotransplantation trials, presumptively attributable to their unique immune properties [12], which provides evidence that cells can be used in various diseases with a low risk. Moreover, the umbilical cords can be easily harvested, and the collection process is noninvasive. Therefore, umbilical cord mesenchymal stem cells (UCMSCs) have been deemed one of the best sources of MSCs in clinical and scientific research.

Kim et al. [13] demonstrated that cord blood-derived endothelial progenitor cells (EPCs) and EPC-derived conditioned medium function equivalently in improving wound healing in diabetic mice. Intriguingly, MSCs can participate in angiogenesis by forming blood vessel around human umbilical cord blood-derived EPCs [14]. Factors secreted by EPCs can stimulate endothelial cell (EC) proliferation but not adhesion, while factors secreted by MSCs can support EC adhesion but not proliferation *in vitro* [15]. The early outgrowth populations of EPCs, including colony-forming unit-endothelial cells and circulating angiogenic cells, are mainly composed of myeloid hematopoietic cells with a proangiogenic secretory phenotype [16–18]. However, the late outgrowth EPCs or blood-outgrowth ECs, also named as endothelial colony-forming cells (ECFCs), are different from the early outgrowth CD14^+^/CD45^+^ EPCs and involved in vascular regeneration via a paracrine manner [19]. Previous studies have demonstrated that ECFCs originating from the peripheral blood-derived mononuclear cell express CD34 [20, 21] and are negative for CD14 and CD45. Moreover, according to the expression profiles of endothelial lineage surface markers evaluated by flow cytometry, Tasev et al. found that peripheral blood-derived ECFCs isolated in platelet lysate-containing medium were positive for CD31, CD34, CD144, CD146, CD309, and CD105 and negative for hematopoietic cell surface antigens CD14, CD45, and CD133 [22]. ECFCs can differentiate into ECs, which directly integrate into the vascular network to participate in vascular reendothelialization and promote angiogenesis via autocrine mechanism [23, 24]. The ECFCs obtained from cord or peripheral blood show significant vascularization ability *in vivo* by physically integrating into newly formed blood vessels [17]. Thus, ECFCs have been recognized as the particularly suitable target cells for therapeutic angiogenesis [25]. Furthermore, the proangiogenic capacity of ECFCs is apparently enhanced in combination with stem/progenitor cells like MSCs or adipose-derived stem cells [14]. Thus, a combination of these two cell populations may produce better paracrine effects after transplantation in regenerative medicine. Additionally, our previous study showed that coadministration of MSCs and ECFCs accelerates wound healing in diabetic mice [26]. Based on these aforementioned researches, the present study aimed to observe the effect of cotransplanted MSCs and ECFCs on diabetic wound healing and to explore a potential method for treating chronic wounds in diabetic patients.

## 2. Material and Methods

### 2.1. Study Population

Twelve diabetic patients with foot ulcers were enrolled in a present clinical study conducted by the Third Xiangya Hospital from December 2016 to March 2019. For the five cases with multiple wounds, one or two of the wounds was treated with stem cell injection after debridement, and adjacent or similar wounds were as self-controls. Wound healing rates before and after cell transplantation were compared in the remaining seven patients with only one wound. The grades of wounds were defined according to the Wagner wound classification, which can be categorized into the following six grades: grade 0 (healed or preulcerative lesion), grade 1 (superficial ulcer without penetrating to deeper layers), grade 2 (deeper ulcer reaching the tendon, bone, or joint capsule), grade 3 (deeper tissues involved and abscess formation with osteomyelitis or tendinitis), grade 4 (partial foot gangrene), and grade 5 (whole foot gangrene) [27, 28]. Although this system has been validated and frequently used in different settings [29–32], the description of DFU via the Wagner classification has been considered to be very simplistic and linear with poor specificity, which defines most of the wounds as grades 2 or 3 in clinical practice [30]. In addition, all patients underwent a standardized examination based on the International Working Group on Diabetic Foot Guidance [33].

Our investigation conformed with the principles outlined in the Declaration of Helsinki. According to the policy approved by our local ethical committee at the Third Xiangya Hospital of Central South University, China, all tissue samples were obtained after informed consent. This clinical case study was conducted in accordance with the principles of the Declaration of Helsinki (1996) and good clinical practice guidelines. The protocol, informed consent form, and other study-related documents were reviewed and approved by the Ethics Committee of the Third Xiangya Hospital of Central South University. All patients provided written informed consent.

Inclusion criteria were as follows: (1) all subjects had DFUs with Wagner grades 2–4; (2) after thorough debridement of free, nonviable, hyperkeratinized, and fibrotic tissues, ulcer with an area ranging from 1 cm^2^ to 60 cm^2^ was included. For patients with multiple lesions, one severe lesion was selected as a target lesion for eligibility confirmation and subsequent assessment; (3) type 1 or type 2 diabetic patients aged between 18 and 75 years were eligible for enrollment; (4) all participants voluntarily signed an informed consent form and agreed to comply with all protocol requirements, including self-care of wounds and follow-up requirements; (5) after two weeks of conventional treatment, such as debridement, antibiotic treatment, hydrocolloid dressing, and foot off-loading (foam dressings and custom-made insoles), DFU patients who showed a reduction of less than 20% in ulcer size were selected as experimental subjects; (6) diabetic patients with the ankle-brachial index (ABI) (calculated using the highest ankle systolic pressure) of the affected limb ≥ 0.5 and ≤ 1.3 were enrolled.

Exclusion criteria were as follows: (1) patients with a significant acute cardio-cerebrovascular event within three months prior such as acute myocardial infarction and acute stroke, (2) presence of severe renal insufficiency (creatinine clearance < 30 mL/min/1.73 m(2)), (3) presence of previously diagnosed or suspected malignancy, (4) corticosteroids and immunosuppressants use, (5) malignant wounds, (6) presence of mental disability that compromises the therapeutic effect or accuracy of assessment, (7) women who are planning to get pregnant during the study period or have already become pregnant, and (8) sinus tracts that could not be removed by debridement.

### 2.2. Isolation and Culture of UCMSC and ECFC

Human umbilical cord blood (UCB) and umbilical cord samples from five healthy full-term infants (three boys and two girls) with a gestational age of 39–40 weeks were collected. This experiment was approved by the Research Ethics Committee of the Third Xiangya Hospital, and informed consent of all newborn parents has been obtained.

Fresh human umbilical cords were obtained after full-term delivery and washed with cold Hank's balanced salt solution (Sigma-Aldrich, St Louis, MO, USA). MSCs were isolated from the mononuclear cell fraction of human umbilical cords, as described in [34] and according to the International Society for Cellular Therapy standard criteria for MSC isolation and characterization [35]. Briefly, mesenchymal tissues were cut into pieces of 1–2 mm^3^ and suspended in solution. After centrifugation at 250 × g for 5 min, the fragments adhered to the substrate of culture plates separately, which was followed by incubation in Dulbecco's modified Eagle's medium with low glucose and 10% fetal bovine serum (FBS). About twelve days later, well-developed colonies of UCMSCs appeared. Subsequently, the colonies were trypsinized and cultured on new cell culture flasks for further expansion.

The characteristics of hUC-MSCs were identified by phenotypes and the potential to differentiate into adipocytes and osteoblasts [36]. Flow cytometry analysis for CD14, CD31, CD34, CD44, CD45, CD73, CD90, and CD105 was performed (all from BD Biosciences Pharmingen, San Jose, CA, USA). Adipogenesis was induced by treating cells with 2 mM dexamethasone, 2 mg/L insulin, 0.5 mM 3-isobutyl-1-methylxanthine, and 0.2 mM indomethacin for 14 days and confirmed by intracellular lipid accumulation via Oil Red O staining. Cells were also induced to differentiate using osteogenic supplements (2 mM dexamethasone, 1 M sodium glycerol phosphate, 10 mM vitamin C) for 28 days, and calcium deposition was demonstrated by alizarin red staining. After screening for mycoplasma and endotoxin, UCMSCs at passages 3–5 were collected for engraftment.

ECFCs were isolated from human umbilical cord blood obtained from healthy full-term neonates as previously described [17]. Briefly, blood samples (60–80 mL) collected in heparinized vacutainer tubes were diluted and overlaid onto Ficoll-Paque PLUS (Amersham, Piscataway, New Jersey). Mononuclear cells from the buffy coat retrieved from UCB by low-density centrifugation were resuspended in endothelial growth medium (EGM-2) (Lonza, Walkersville, MD, USA) supplemented with 10% FBS and then seeded at 5 × 10^7^ cells/well onto 6-well tissue culture plates (Costar Corp.) coated with type I rat tail collagen (BD Biosciences, Bedford, MA) at 37°C, 5% CO_2_ in a humidified incubator. After 24 hours of incubation, adherent cells were washed with PBS and cultured with complete EGM-2 medium. Medium was changed daily for the first seven days and then every other day until confluency was reached. Typical cobblestone colonies appeared between day 6 and 8 in culture and were characterized as circumscribed monolayers of cobblestone-shaped cells. After reaching confluency of 70 to 80%, endothelial cells were detached with 0.25% trypsin-ethylenediaminetetraacetic acid (EDTA) (Invitrogen) and subsequently resuspended in complete EGM-2 medium and cultured in flasks or dishes coated with type I rat tail collagen. Isolated ECFCs of passages 3–5 were for cell assays and transplantation. ECFC and MSC were cultured separately in appropriate media to maintain their characteristics. Adherent cells were trypsinized before coinjection.

### 2.3. Cell Administration at the Wound Site

Prior to cell injection, wound areas of necrotic and devitalized soft tissues were surgically debrided. Cells were harvested by trypsinization and washed several times with phosphate-buffered saline and were subsequently filtered through a 0.22 *μ*m filter into a sterilized container. 2–5 mL mixture of UCMSCs and ECFCs (0.2–1 × 10^7^ cells, 2 : 1) was injected through a 23-gauge needle, both in the center of the wound area and along the wound border. Following cell injection, 2–4 mL hydrogel-based on 0.5% (*w*/*v*) hyaluronic acid (HA) was applied to the wound and formed a clot on the surface, which was further covered by Vaseline oil gauze. The hydrocolloid dressings were used to cover the wounds until healing or the end of the study. Changing dressings and taking photographs of wounds were performed every 3–4 days during the first four weeks. The formation of granulation tissue and closure of wound was closely observed. After discharge from the hospital, the patients were followed up regularly for ulcer recurrence and any other possible complications.

### 2.4. Estimation of Clinical Outcome

The advancement of wound healing was assessed through clinical observation of the wound during dressing changes and at follow-up, recording pictures and assessment of different parameters related to wound healing. Wounds were considered healed when complete reepithelialization was observed. Wounds were considered improved when wound size had decreased. The wound healing area was assessed once every 3–7 days after the procedure until complete wound closure was observed and compared with the control wound or preimplant baseline values of the same wound. The wound area was measured by tracing the wound margin on the photograph and calculating the pixel data using the ImageJ software (National Institutes of Health, Bethesda, MD). The time to complete wound closure was assessed (*n* = 12). The follow-up study was undertaken for up to twelve weeks. The wound area after four weeks and complete healing time were collected. Reepithelialization rate (cm^2^/week) and the healing rate within the first week (%) were calculated using the following formulae commonly used for the assessment of wounds [37–39].

(1) $$\begin{array}{c} \text{Reepithelialization rate }\left({\text{cm}}^{2}/\text{week}\right)=\frac{\text{Epithelium area }{\text{day}}_{\text{n}1}-\text{Epithelium area }{\text{day}}_{\text{n}2}}{{\text{day}}_{\text{n}1}-{\text{day}}_{\text{n}2}}\\ \text{Wound healing }\left(\%\right)=1-\left(\frac{\text{Open wound area }{\text{day}}_{n}}{\text{Total wound area }{\text{day}}_{0}}\right) \end{array}$$

### 2.5. Statistical Analysis

IBM SPSS Version 25.0 for Windows and GraphPad Instant statistical package (GraphPad Software Inc.) were used for statistical analysis and visualization, respectively. The data were presented as mean ± standard deviation (SD). The Kolmogorov-Smirnov test was used for normality analysis. Normally distributed variables were analyzed using paired sample *t* test. The data that were not normally distributed were analyzed using the Wilcoxon's signed ranks test. A two-sided *P* < 0.05 was considered statistically significant.

## 3. Results

The demographic details of the patients are shown in Table 1. Twelve patients (seven males) with DFUs were recruited for this study. The mean ± SD of age was 56.58 ± 10.09 years. The mean ± SD of diabetes duration and wounds duration was 12.17 ± 9.46 years and 2.45 ± 3.42 months, respectively. All participants were with an average ABI of 1.18 ± 0.47 (Table 1).

### 3.1. The Combined Use of UCMSCs, ECFCs, and HA Accelerates Wound Healing in Five Diabetic Patients with Multiple Wounds

**Figure 1:**
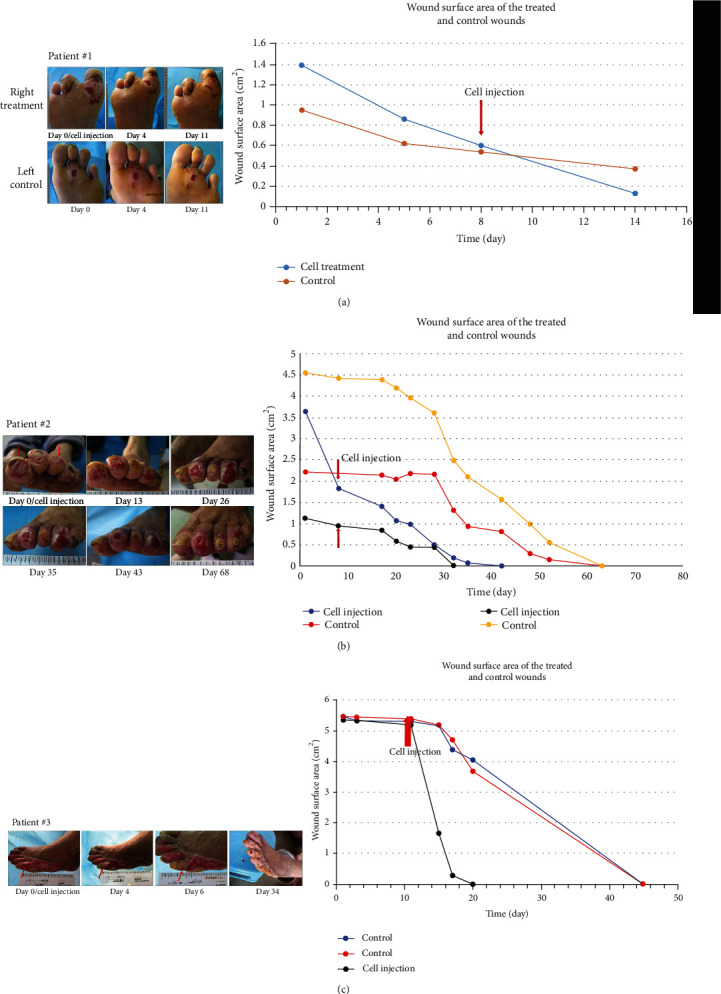
Photographs of treated ulcer (right foot) and control ulcer (left foot) over time with depiction of wound surface area of patient #1 (a). Patient #1 had two wounds in both foots; the right foot was injected cells and the left foot as control. The healing rate of the wound injected cells was obviously faster than control wounds. Photographs of treated ulcer (the first and third toes) and control ulcer (the other two toes) over time with depiction of wound surface area of patient #2 (b). Patient #2 had four wounds in his left foot; the first and third toes were injected cells (red arrow) and the other two toes as control. The healing rate of the toes injected cells was obviously faster than control wounds. Photographs of treated ulcer (the fifth toe) and control ulcer (the other two toes) over time with depiction of wound surface area of patient #3 (c). Patient #3 had three wounds in his left foot; the fifth toe was injected cells (red arrow) and the other two toes as control. The wound was covered with white epithelium at the fourth day after cell treatment. The healing rate of the fifth toe was obviously faster than control wounds.

For patients #1–5 with multiple wounds as self-control, the healing rate and reepithelialization rate of wounds treated with cell injection were significantly faster than control wounds within the first week (Table 2). The first three patients all showed a significant increase in wound healing rate after cell therapy (Figure 1). Within the first week after cell application, reepithelialization rate and healing rate were about six-fold and two-fold more than those of the control group. Moreover, the wounds of three patients in the treatment group were completely closed within four weeks, while only one patient in the control group showed complete wound healing during this study. The mean time required for complete wound healing was significantly shorter in the treatment group than in the control group (*P* = 0.032, Table 2).

### 3.2. The Reepithelialization and Healing Rates after Cell Transplantation Were Significantly Faster Than Those before Injection in the Seven Diabetic Foot Patients with Only One Refractory Wound

**Figure 2:**
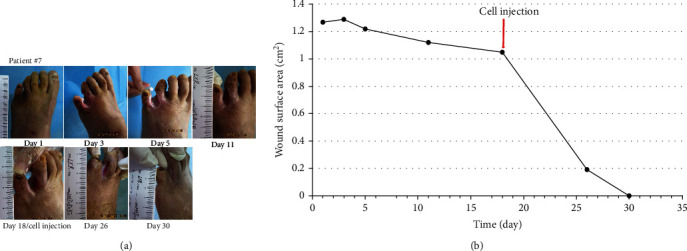
Photographs of ulcer before and after treatment (a) with depiction of wound surface area (b). Patient #7, a 64-year-old woman with a 5-year history of type 2 diabetes, presented with ulcer and infection on her left foot (Wagner 3) for 2 months.

**Figure 3:**
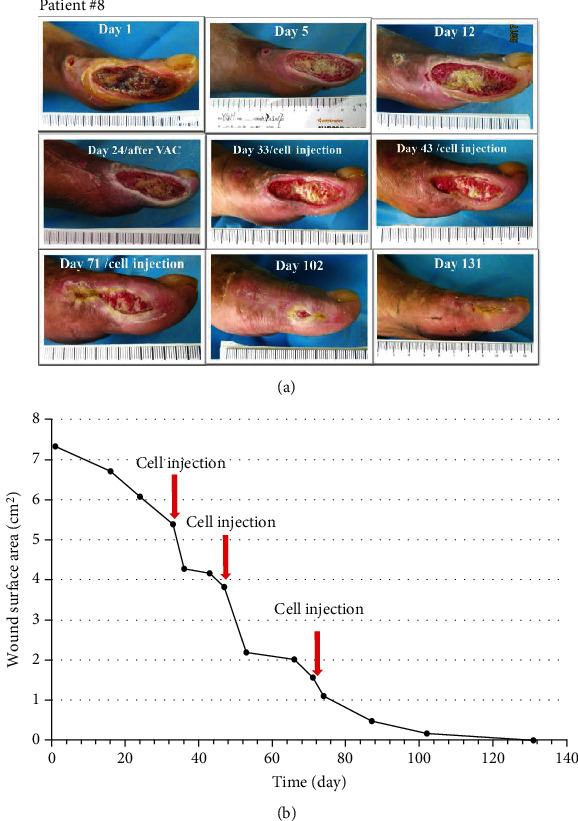
Photographs of ulcer before and after treatment (a) with depiction of wound surface area (b). Patient #8, a 64-year-old man with an 11-year history of type 2 diabetes, had the wound on his left foot for one month (Wagner 3); moreover, the wound area was 7.33 cm^2^ with bone exposure after debridement of the first toe.

**Figure 4:**
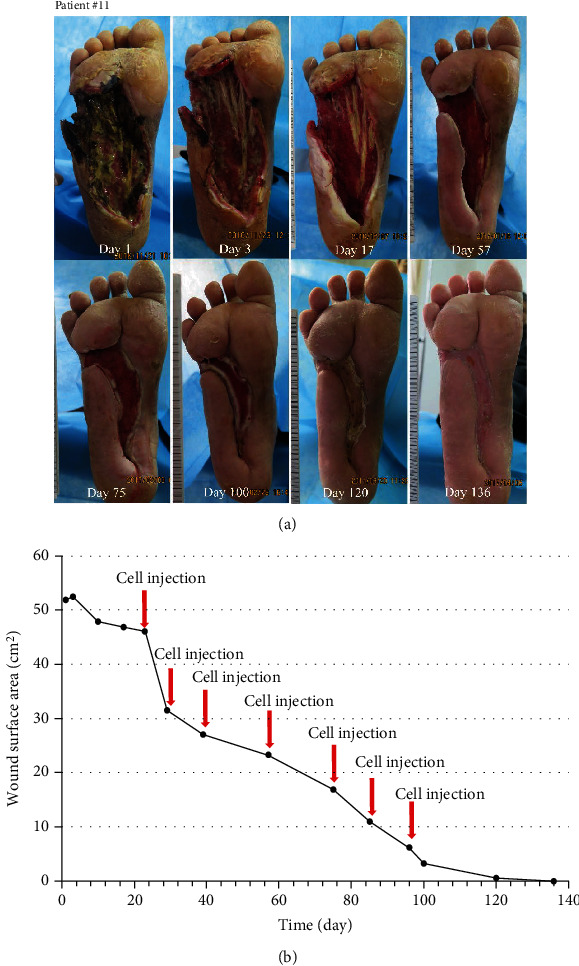
Photographs of ulcer before and after treatment (a) with depiction of wound surface area (b). Patient #11, a 47-year-old man with a 10-year history of type 2 diabetes, presented with infection of his right foot (Wagner 4), which was present for two months; the wound area was 52.39 cm^2^ after debridement.

**Figure 5:**
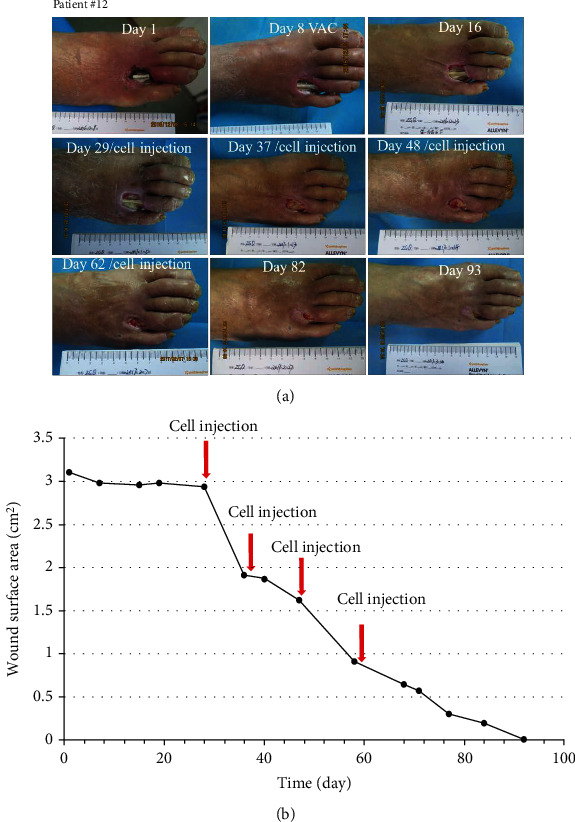
Photographs of ulcer before and after treatment (a) with depiction of wound surface area (b). Patient #12, a 69-year-old man with a 30-year history of type 2 diabetes, presented with infection of his right foot and exposed bones and tendons (Wagner 3), which was present for one week; the wound area was 3.10 cm^2^ after debridement of the foot.

Seven patients with refractory wound, prior to the application of cultured cells, the ulcer was standard treatment for at least two weeks. During this time, the ulcer reduction in size is very slow (healing rate 9.0%); when MSCs and ECFCs were injected at the site of wounds, wound closure was significantly accelerated (healing rate 44%); results are presented in Table 3. The mean complete healing time after cell injection was 42.57 ± 31.38 days. Three patients were completely closed after four weeks, and there was a significant wound reduction after treatment (*P* < 0.05) in the remaining patients. Pictures of the wounds, before and after treatment of patients #7, #8, #11, and #12, are presented in Figures 2–5, respectively.

## 4. Discussion

With the high rates of disability and death, chronic DFU is one of the most refractory complications of diabetes mellitus and largely affects the quality of life and life expectancy of patients [40]. Despite the widely taken clinical measures, including debridement, decompression, antibacterial therapy, neurotrophic improvement, and revascularization, the risk of amputation still remains high in patients with DFU [41]. In China, the amputation rate of diabetic patients was 19.03% in 2015 [42]. Furthermore, in recent years, new techniques including autologous platelet-rich gel [43] and marrow stem cells [44, 45] have achieved good therapeutic effects in treating chronic wounds of DFU.

The potential of self-renewing and multidifferentiation are two main features of stem cells, which make stem cell therapy a novel application for tissue regeneration. Nowadays, a growing number of diseases can be improved via stem cell transplantation, such as congenital cataract [46], diabetic retinopathy and keratopathy [47], serious skin burns [48], and DFU [49]. ECFCs, progenitor cells committed to endothelial lineages, own robust vasculogenic properties [17, 50]. Importantly, ECFCs are also capable to form entirely new vascular structures and repair damaged host vasculature under the context of *in vivo* transplantation, which indicates their potential importance in treating cardiovascular diseases [51, 52]. However, the limitation in their use as an autologous cell therapy product still exists due to the small number and poor expansive property of ECFCs isolated from human adults [53, 54]. Furthermore, the immunogenicity of ECFCs is another important theoretical limitation in allogeneic use, and it is difficult to access abundant autologous ECFCs for cellular therapy. Therefore, strategies to improve the therapeutic potential of ECFCs are urgently needed.

Recent experimental and clinical studies with autologous MSCs have identified numerous mechanisms in promoting wound healing, including anti-inflammatory, antiapoptotic, and angiogenetic properties [55, 56]. Accumulating evidence has shown that MSCs might enhance wound healing [57–59] and serve as a cell source in many tissue engineering applications including bone regeneration [60], neurogenesis [61, 62], and DFU [6, 63]. Moreover, MSCs have been described to support ECFC-mediated angiogenic processes in various matrices [64]. MSCs are widely known for its ability to accumulate to sites of injury *in vivo* and manipulate the microenvironment via paracrine and immunomodulatory capacity, thereby assisting host tissue regeneration and promoting angiogenesis [65, 66]. The synergistic effects of MSC exerting onto ECFC could either through coculturing or coinjection [67, 68]. Besides, this joint effect led to increased human ECFC survival *in vivo* and increased vessel formation in immunocompetent mice; furthermore, levels of engraftment and vessel formation were higher when MSC and ECFCs were combined in immunodeficient animals, suggesting the benefits of adding immunosuppression treatment to the cellular therapy [68]. However, the interactions between MSC and ECFC in DFU still remain unclarified. Thus, we examined whether coadministration of ECFCs and MSCs could accelerate wound healing in diabetic patients. In the present study, considering that wound healing is related to various factors such as blood glucose level, nutritional status, and the degree of nerve and vascular lesions that cause foot ulcers, we selected five patients with multiple ulcers, of which 1–2 ulcers were used as the cell intervention group and others as the control group. The healing rate of ulcers treated with UCMSC and ECFC was significantly higher than that in the control group. The other seven DFU patients initially responded poorly to traditional treatments, including debridement, offloading, and complementary therapies (such as antibiotic therapy, controlling blood glucose level, and irrigation of wound with normal saline and dressings). However, after treatment of a combination therapy of UCMSCs, ECFCs, and HA, patients showed improved wound status, and all wounds were completely closed within twelve weeks, although some wounds were still with exposed bones and in huge size.

In the present study, the topically applied HA-based hydrogel might also facilitate the ulcer healing process. As the main component of the extracellular matrix [69], HA presents unique characteristics for its application in regenerative medicine. HA has been widely used in many medical devices; furthermore, the advanced wound care device based on HA is indicated for treating partial to full-thickness posttraumatic, postsurgical, or deep-chronic wounds, including vascular ulcer and DFU [70]. Recent *in vivo* studies in animal models have tested hydrogel composites of human UCB-MSCs mixed with 4% sodium hyaluronate have shown an improvement in the histologic appearance of the repaired articular cartilage tissue [71, 72]. Thus, though stem cells possess the capacity to migrate to the site of injury, HA hydrogels combined with drugs or growth factors were developed in order to guide endogenous stem cells towards a specific site or injured area, which can largely promote the tissue regeneration [73]. Rho et al. showed that HA-based nanoparticles could suppress the inflammation of the adipose tissue in diabetes due to a decrease in macrophages and proinflammatory cytokines [74]. Therefore, previous reports mainly focused on cartilage and skin regeneration have shown that the combination of HA and stem cells is safe and improves pain reduction and therapeutic efficacy through enhancing cell survival and tissue restoration. In this study, we also found that topically applied HA combined with coadministration of ECFC and MSC promoted the wound healing of type 2 diabetic patient with nonhealing foot ulcers. However, a major limitation to prove the specific role of HA in diabetic wound healing here is the lack of wound groups with only cell injection treatment. Thus, we will further perform a more comprehensive research about HA combined with cell therapy in the diabetic murine models.

Various methods have been applied for the management of DFUs including antibiotics, hyperbaric oxygen, negative pressure, neuropathic drugs, anti-inflammatory drugs, tissue regeneration products, autologous platelet-rich plasma, and recombinant growth factors [75]. Even though some of these treatments might be effective, no single treatment can be accepted as a standard therapy for all diabetic patients so far [75], which can be explained by the biochemical and physiological variations of the diseases. Furthermore, the gold standard treatment of huge wound used in the clinic is the autologous split-thickness skin graft, which involves removing a piece of skin from a secondary surgical site for the patient, stretching the skin, and reapplying the graft on the wound. Although this treatment yields a reasonable clinical outcome, a secondary surgical site in diabetes will be created. Noteworthy, two patients with large wounds in present study were healed without skin grafting, which suggested this graft could be used in diabetic large skin wounds. Wound healing requires a well-orchestrated integration of the complicated biological and molecular events of cell migration, proliferation, and extracellular matrix deposition and remodeling [76]. In this study, combinations of UCMSCs and ECFCs transplantation were used to treat twelve patients with refractory DFU. No obvious adverse reactions or residual pathological syndromes appeared during transplantation. Toxic reactions of UCMSCs and ECFCs were not detected during the 1-year follow-up (data not shown). However, because of the small number of patients enrolled in the present study, our results need to be validated in large clinical trials in the future. Another limitation is that we only used the Wagner classification system, which provided little information about the ulcer size, degree of polyneuropathy, and wound infection status. The S(AD)SAD and the PEDIS (perfusion, extent, depth, infection, and sensation) classification systems can be more accurate in evaluating the degrees of lower extremity ischemia, soft tissue infection, and neuropathy [77, 78]. In addition, as a validated method to describe the grade and stage of DFU, the University of Texas (UT) diabetic wound classification system has been shown to be more closely associated with an increased risk of amputation and a better predictor of outcome compared to the Wagner system [29]. Furthermore, the interobserver agreement indicated that the Wagner and UT systems did not seem to be useful as a single instrument and should be used in combination with additional clinical information to avoid false interpretations [28].

## 5. Conclusion

In this report, we firstly compared the healing rate of two separate wounds in the same patient: one treated with coadministration of MSCs and ECFCs as well as topically applied HA, while the self-control wounds were only applied with hydrocolloid dressing. In addition, the difference between the area of wound before and after treatment in the same patient with only one ulcer was also estimated. The treated wound showed accelerated healing in comparison to the control wound or the same wound before cell injection therapy. Therefore, the combination therapy of UCMSCs, ECFCs, and HA accelerates the healing of diabetic refractory wounds, which might potentially reduce the healthcare cost and surgical burden.

## Figures and Tables

**Table 1:** Baseline characteristics of participants.

	Total
n	12
Male	7 (58.33)
Age (years)	56.58 ± 10.09
Duration of diabetes (years)	12.17 ± 9.46
Duration of wound (months)	2.45 ± 3.42
ABI	1.18 ± 0.47

The results are presented as mean ± SD or *n* (%). SD: standard deviation; ABI: ankle-brachial index.

**Table 2:** The comparison of the treatment group and control group among five patients with multiple wounds.

	Treatment group	Control group	t*/*z	P
Wound area after debridement (cm^2^)	6.96 ± 8.90	4.03 ± 2.98	-0.674^b^	0.500
Reepithelialization rate within the first week (cm^2^/week)	2.60 ± 2.98	0.37 ± 0.35	-2.032^b^	0.042∗
The healing rate within the first week (%)	0.61 ± 0.35	0.21 ± 0.19	3.344^a^	0.029∗
Wound area after four weeks (cm^2^)	1.83 ± 4.01	1.99 ± 1.97	-0.365^b^	0.715
Complete healing time after injection (day)	24.40 ± 18.76	37.60 ± 18.72	-3.217^a^	0.032∗

The healing rate and reepithelialization rate of wounds treated with cell injection were significantly faster than the control wounds within the first week, also with much shorter complete healing time. The data are presented as mean ± SD, ∗*P* < 0.05 vs. control wounds. ^a^Paired t test, ^b^Rank sum test.

**Table 3:** Reepithelialization rate, the healing rate within the first week, wound area before debridement, and four weeks after cell injection among seven patients with one refractory wound.

	Before cell injection	After cell injection	z	P
Reepithelialization rate within the first week (cm^2^/week)	0.58 ± 0.52	3.61 ± 5.96	-2.366^b^	0.018^∗^
The healing rate within the first week (%)	0.09 ± 0.06	0.44 ± 0.18	-2.366^b^	0.018^∗^
Wound area (cm^2^)	11.36 ± 18.31	4.28 ± 9.17	-2.366^b^	0.018^∗^

The reepithelialization rate and healing rate of wounds given cell injection were significantly faster than before cell injection within the first week (mean ± SD), ^∗^*P* < 0.05 vs. wound before cell injection. ^b^Rank sum test.

## Data Availability

All datasets generated for this study are included in the article.

## Additional Points

*Summary*. Wound care represents a significant socioeconomic burden, with over half of chronic wounds taking up to a year to heal. Measures to accelerate wound healing are beneficial to patients and reduce the cost and burden of wound management. Coadministration of mesenchymal stem cells (MSCs) and umbilical cord blood-derived endothelial colony-forming cells (ECFCs) is an emerging option for autologous skin grafting in the outpatient setting to improve wound healing. Although several case series have previously reported good clinical outcome with coadministration of MSCs and ECFCs, the healing rate in comparison to conservative wound management is still unknown. In this report, we compare the weekly healing rate of two separate wounds in the same patient, one treated with coadministration of MSCs and ECFCs as well as topically applied hyaluronic acid, while the self-control wounds were only applied with hydrocolloid dressing. The treated wound showed accelerated healing, and the average healing time of the treated wound was about 40% faster compared to the control wound. Therefore, the combination therapy of UCMSCs, ECFCs, and hyaluronic acid accelerates the healing of diabetic refractory wounds, which might potentially reduce the healthcare cost and surgical burden.
